# Surface Modification of Nano-Hydroxyapatite/Polymer Composite for Bone Tissue Repair Applications: A Review

**DOI:** 10.3390/polym16091263

**Published:** 2024-05-01

**Authors:** Shuo Tang, Yifei Shen, Liuyun Jiang, Yan Zhang

**Affiliations:** 1 National & Local Joint Engineering Laboratory for New Petro-Chemical Materials and Fine Utilization of Resources, College of Chemistry and Chemical Engineering, Hunan Normal University, Changsha 410081, China; 2 Key Laboratory of Chemical Biology & Traditional Chinese Medicine Research (Ministry of Education, China), College of Chemistry and Chemical Engineering, Hunan Normal University, Changsha 410081, China; 3 Key Laboratory of Light Energy Conversion Materials of Hunan Province College, Hunan Normal University, Changsha 410081, China

**Keywords:** nano-hydroxyapatite, surface modification, polymers, bone repair

## Abstract

Nano-hydroxyapatite (n-HA) is the main inorganic component of natural bone, which has been widely used as a reinforcing filler for polymers in bone materials, and it can promote cell adhesion, proliferation, and differentiation. It can also produce interactions between cells and material surfaces through selective protein adsorption and has therefore always been a research hotspot in orthopedic materials. However, n-HA nano-particles are inherently easy to agglomerate and difficult to disperse evenly in the polymer. In addition, there are differences in trace elements between n-HA nano-particles and biological apatite, so the biological activity needs to be improved, and the slow degradation in vivo, which has seriously hindered the application of n-HA in bone fields, is unacceptable. Therefore, the modification of n-HA has been extensively reported in the literature. This article reviewed the physical modification and various chemical modification methods of n-HA in recent years, as well as their modification effects. In particular, various chemical modification methods and their modification effects were reviewed in detail. Finally, a summary and suggestions for the modification of n-HA were proposed, which would provide significant reference for achieving high-performance n-HA in biomedical applications.

## 1. Introduction

**Figure 1:**
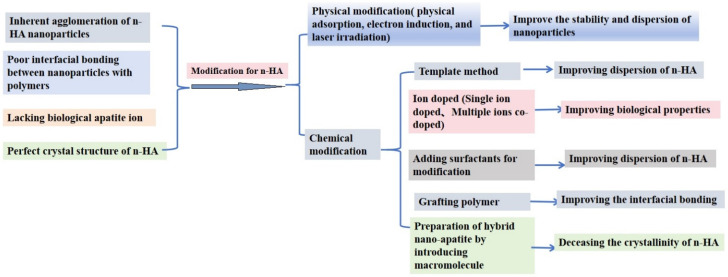
Modification strategies for n-HA.

Bone defects caused by trauma, infections, and bone tumors are very common. Conventional autologous or allogeneic bone grafting has its own limitations, while artificial bone grafting is currently the most popular treatment for all types of bone defect repair, including dental bone implantation and cranial defect repair in neurosurgery. As is known to us, bone matrix is an extracellular matrix in bone tissue that has undergone calcification, consisting of 65% inorganic phase and 35% organic phase [1]. Nano-hydroxyapatite (Ca_10_(PO_4_)_6_(OH)_2_, n-HA) is the major inorganic constituent in human hard tissue, and its chemical composition and structure are very similar to those of biological bones and enamel, so n-HA is known as a highly biocompatible, bioactive, osteoconductive, non-toxic, non-inflammatory, and non-immunogenic agent [2,3,4]. The preparation methods of n-HA mainly include the hydrothermal method, chemical precipitation method, microwave solid phase method, sol–gel method, spontaneous combustion method, and electrochemical deposition method [5,6,7]. Among the preparation methods, the chemical precipitation method is the most commonly used preparation method, which is a mild experimental approach without expensive equipment. However, the synthesized n-HA is used alone in orthopedic materials; it exhibits some inherent defects in clinical applications, such as high brittleness and in vivo difficulty in degradation, and its biological activity needs to be improved. To compensate for these deficiencies, it is simple to combine n-HA with degradable polymers so as to obtain high-performance bone materials [8]. However, it has been shown that n-HA is difficult to disperse uniformly in the polymer due to its inherent agglomeration of n-HA nano-particles, and there exists poor interfacial bonding between nano-particles and polymers via physical blending, which could lead to poor mechanical properties. In addition, the biological apatite usually contains a small amount of carbonate, fluorine, silicon, magnesium, sodium, citric acid, etc., so there are some differences between the synthesized n-HA and the biological apatite, resulting in its insufficient osteogenic activity, so it is difficult to obtain the vascularized bone formation and achieve good bone integration. Moreover, conventional n-HA is difficult to biodegrade in vivo due to its perfect crystal structure of n-HA. Therefore, it is very necessary to carry out the modification of n-HA so as to obtain the n-HA/polymer nano-composite with the aim of expanding its application in the biomedical field. To enable readers to have a clearer understanding of the reasons and modification strategies for n-HA, the logic behind the surface modifications of n-HA is summarized (shown in Figure 1).

## 2. Physical Modification

The methods of physical modification include physical adsorption, electron induction, and laser irradiation, and the main purpose is to improve the stability and dispersion of nano-particles. Aronov D et al. [9] reported on surface free energy modulation of a hydroxyapatite-coated titanium femoral implant via low-energy electron irradiation. The selective bacterial adhesion in combination with the ability to define the surface energy properties suggests that this method opened an avenue for the protection of implants from bacterial infections. Queiroz AZ et al. [10] used a KrF excimer laser with a wavelength of 248 nm and a pulse duration of 30 ns to modify the surface of n-HA, and a series of characterizations showed that the surface modification with the laser could increase the surface area of n-HA, making it a promising technology with for improving reactivity and drug-delivery ability. Physical modification had been favored by researchers due to its advantages such as easy handling, low production cost, and no pollution. However, the organic molecules were bound to the surface of n-HA particles by a non-covalent bond, so the physically adsorbed organic molecules could be easily washed out with the body fluid. While the chemical modifier is bound to the surface of the HA particles via a chemical bond, it is more stable than physical modification, so the chemical modifier is used more frequently.

## 3. Chemical Modification

Chemical modification is preferred for the improvement of the morphology, crystal structure, and surface properties of n-HA via a chemical reaction. According to the different reaction mechanisms, it can be divided into several types: template method, doping method, surface grafting of small molecules or polymers, and hybrid macromolecules.

The modification methods of n-HA was shown in Table 1, and the comparison of the different chemical modification methods was given in Table 2.

### 3.1. Template Method

**Figure 2:**
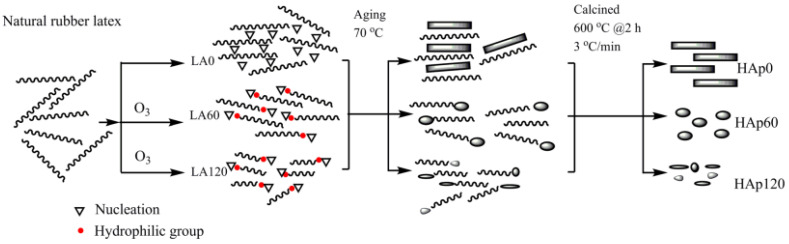
The formation mechanism of synthesized HAp templated by ozonolyzed natural rubber latex.

The template method involves the interaction between the precursor of the synthesized n-HA particles and an organic substance (template) with a certain morphology or structure, so that the generated n-HA is covered on the surface of the template or embedded inside it to form a composite, and the modified n-HA with different morphologies or structures is obtained by removing the template. Zhou H et al. [11] reported a one-step hydrothermal method to synthesize mesoporous HA with the assistance of a cost-effective template vitamin C. The mesoporous HA exhibited enhanced adsorption of the model drug doxorubicin compared to conventionally synthesized HA. Aguilar AEM et al. [12] synthesized n-HA via chemical precipitation using Euclea Natalensis root extract as a template. The results showed that n-HA from the green route presented a spherical-like shape with a smooth surface, and the surface of n-HA without the green template was covered with nanogrooves. Utara S et al. [13] successfully synthesized HA by means of the sol–gel method in the presence of ozonolyzed natural rubber latex templates of various molecular weights, and the formation mechanism of synthesized HA templated by ozonolyzed natural rubber latex is shown in Figure 2. The results showed that the molecular weight, as well as the functionality of the biomacromolecule template, influenced the phase crystallinity and morphology of synthesized HA. From the literature review, it can be concluded that the template had a great effect on the morphology of HA, which is suitable for obtaining HA nano-particles with various morphologies.

### 3.2. Ion Doped

#### 3.2.1. Single Ion Doped

Doping HA with foreign ions is becoming more and more popular as a chemical method to enhance its performance and endow it with new characteristics [14]. Some cations, such as M^2+^, can be easily exchanged with Ca^2+^ in HA to form an apatite-based solid solution. Some anions, such as Cl^−^ and F^−^, can be easily replaced with OH^−^ in HA to form a solid solution of chlorapatite or fluorapatite with HA, which can change the surface properties of n-HA. Therefore, ion doping is achieved in the preparation of n-HA by adding corresponding ions to the reactants, which improves the surface properties of n-HA. Ma P et al. [15] applied strontium-substituted hydroxyapatite (Sr-HA) nano-particles on the surface of polyethylene terephthalate (PET) artificial ligament, and the results showed that the prepared coating significantly improved surface hydrophilicity and promoted osteogenic differentiation and bone integration to repair ligament damage in rabbits, thus providing a potential method for the use of PET artificial ligaments modified with Sr biomaterials to reconstruct ACL. Besides strontium doping, magnesium doping also has similar effects because they belong to the same main group of elements. Zhao SF et al. [16] also confirmed that Mg-n-HA surface coating could better promote the differentiation of somatic cells before osteogenesis compared to n-HA coating in vitro, and it improved the osseointegration of implants more outstandingly at the early stage of bone healing in vivo. Garbo C et al. [17] synthesized a new porous HA (HAP-Zn) with zinc content ranging from 0.2 to 10 wt% by coprecipitation in the presence of the surfactant L-asparagine and found that its pore size distribution and morphology were controllable, which could be used in orthopedic surgery, especially in the treatment of osteoporosis and as a bone substitute, as well as in dentistry for the remineralization of tooth enamel.

#### 3.2.2. Multiple Ions Co-Doped

To obtain better surface properties of n-HA, a multiple-ion co-doping method has been proposed. Yilmaz B et al. [18] investigated the co-doping of different ions and concluded that when two or more of these ions were doped together, the multiple effects would not be a simple combination of individual contributions as the doping elements directly changed the atomic structure of the doped HA. Predoi D et al. [19] doped a silver and zinc HA coating into a chitosan matrix composite (Ag-Zn-HAp/CS) via the dip-coating method, and the results demonstrated that the presence of Ag-Zn-HAp/CS composite suspension and coating did not affect the morphology of cells and showed good antibacterial performance. Lavanya P et al. [20] prepared copper and manganese mineral-substituted HA (Cu-Mn-HA) and Cu-Mn-HA/chitosan (CTS)-polyvinylpyrrolidone (PVD) via sol–gel and solvent casting techniques, respectively. The results showed that 30% Cu-Mn-HA in CTS-PVD had superior mechanical, physical, and chemical properties, and promoted the deposition of bone-like apatite faster than the biological composites with 0, 10 and 20 wt% Cu-Mn-HA/CTS-PVD, so 30 wt% Cu-Mn-HA/CTS-PVD could be used for bone regeneration. Dittler ML et al. [21] have investigated bioactive glass (BG)-based scaffolds of 45S5 composition covered with hydroxyapatite nano-particles loaded with Mg^2+^, Zn^2+^ and both Mg^2+^ and Zn^2+^ ions (noted as HA-BG, Zn-HA-BG, Mg-HA-BG, and Mg-Zn-HA-BG scaffolds). The results showed that nano-crystalline Mg-Zn-HA coatings enhanced the biological performance of standard scaffolds of 45S5 BG composition, suggesting that Mg-Zn-HA-coated scaffolds were attractive systems for bone tissue engineering.

Based on the analysis of the results, we conclude that it would be a better strategy to incorporate multiple ions into n-HA, which would be more beneficial in improving the biological properties of bone materials.

### 3.3. Adding Surfactants for Modification

**Figure 3:**
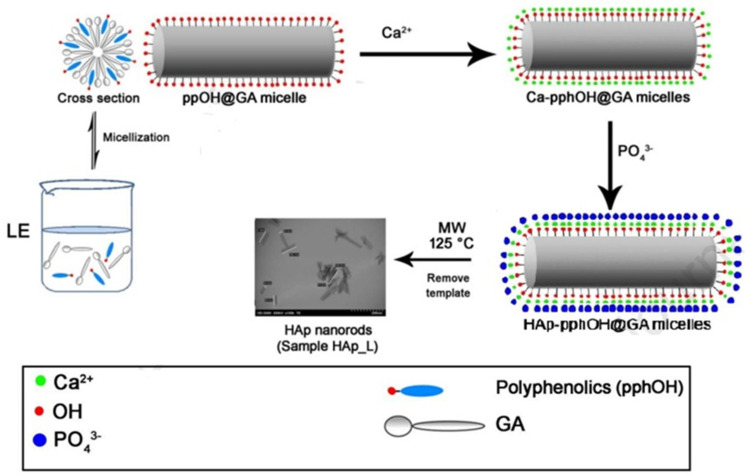
Nano-rod micelles of licorice root extract as a novel green template for the formation of uniform.

The molecules of surfactants have two functional groups with different solubilities or polarities, namely, lipophilic groups (non-polar groups) and hydrophilic groups (polar groups). The surfactants ensure that the nano-particles remain in a stable monodisperse state in the dispersion medium through the adsorption of their groups on the particle surface and changes the surface state of the nano-particles. The surface polarity of n-HA particles is of great importance. When they are modified with surfactant, the polar groups are prone to form strong bonds on their surface. Pang GH et al. [22] explored the surface of n-HA coated with polyethylene glycol, polyvinyl alcohol, and stearic acid and found that the type of surface modifier and the concentration of the active ingredient had a significant effect and a certain selectivity on the particle size, and polyethylene glycol with a concentration of 5% was the best modifier for HA, exhibiting the best dispersibility. Wang SH et al. [16] used stearic acid to coat the surface of HAp through a high-pressure reactor. After modification, the diameter of HAp particles increased and the interfacial compatibility between PLA and HAp was improved; it promoted crystallization, refined the particle size, and led to the evolution of PLA composite from brittle fracture to ductile fracture such that the thermal deformation temperature, tensile strength, and impact strength were significantly increased. Ma TY et al. [24] adopted the hydrothermal method to synthesize well-dispersed HA nano-rods with different morphologies in the reaction system of oleic acid, ethanol, and water and conducted a comparative study on the auxiliary modification effect of surfactants. It was found that the selected surfactants, such as cetyltriethylammonium bromide (CTAB) and sodium dodecyl sulfate (SDS), played an important role in the formation of uniform HA nano-rods. Wang WY et al. [25] prepared high-purity glycine-modified n-HA (HAP-Gly) powder via co-titration with calcium hydroxide, phosphoric acid, and Gly as raw materials because Gly had a certain influence on the crystallization performance of HAP, and the diffraction peak of the modified HAP was significantly broadened. HAP-Gly was a crystal cluster–rod structure with a length of about 50–130 nm and a diameter of about 5–15 nm. Cytotoxicity analysis revealed that it had no cytotoxicity. Yin YJ et al. [26] also compared the changes in the adsorption performance of n-HA after modification with surfactants. Anionic surfactant sodium dodecyl benzene sulfonate (SDBS) was selected for the modification of n-HAP, and it was found that the adsorption capacity of Cd^2+^ after modification was significantly higher than before due to the inhibition of aggregation, which increased the specific surface area, and the introduction of new functional groups provided more sites for the adsorption of Cd^2+^. Lin DJ et al. [27] synthesized HA with the assistance of cationic, anionic, non-ionic, and zwitterion templates. It was found that the uncalcined rod-shaped HA synthesized with non-ionic templates at pH 4 showed excellent cell viability, while anionic, cationic, and non-ionic surfactants showed biocompatibility only after calcination. At pH 9, the non-ionic and un-calcined zwitterion-assisted rod HA showed excellent biocompatibility. Chen RG et al. [28] prepared HA crystals with an arambola-like structure via supersaturated urea-assisted solvothermal synthesis using a dual surfactant. By adjusting the dual surfactant of Na_2_EDTA and stearic acid and the reaction time, the product morphology could be well customized, including microhexagonal prisms, carambola-like structures, and microspheres. Na_2_EDTA had a slight inhibitory effect on the formation of HA, and stearic acid adsorbed onto the surface of HA to form a long chain layer and act as a mechanical barrier, indicating its excellent dispersibility. Zhang SH et al. [29] proposed a method to synthesize dandelion-like HAP cells using an environmentally friendly rosin-based phosphate diester surfactant DDPD as a new phosphorus source, template, and crystal growth control agent. The results showed that the prepared samples exhibited good cell compatibility. Ashraf FA et al. [30] synthesized hexagonal HAp nano-rods in the presence of licorice root extract (LE) via a microwave hydrothermal synthesis route at 125 °C, where LE was used as a green organic template (or biological template), and the crystals displayed uniform morphology and high crystallinity without containing carbonates, whose Ca/P atomic ratio was close to stoichiometric values, confirming that it was a new environmentally friendly green synthesis route (as shown in Figure 3). These HAp nano-rod products using licorice and LE as templates could be widely used in many biomedical fields, such as bone repair, drug delivery, and dental repair.

Sezer D et al. [31] synthesized HAP using templates such as the modification of bromide CTAB, Pluronic hexadecyltrimethylammonium^®^P-123 (P123), and Pluronic™ F-127 (F127) via the chemical precipitation method. The results showed that the surfactant modified with CTAB-HAP had the highest adsorption performance, which was suitable as an alternative carrier for ASA adsorption and controlled release. For this, it can be seen that the design of surfactant components should initially be based on the following two principles: firstly, the anchoring group with anchoring adsorption has an effect on the surface of HAP particles; second, a solvation chain with sufficient length could form stable n-HA particles through three-dimensional obstacles and form an affinity with the solvent. Based on the above design principles, it was important to select appropriate surfactants for the surface modification of n-HA particles.

However, research has shown that the addition of surfactants, such as polyvinylpyrrolidone (PVP), chondroitin sulfate (ChS), aspartic acid (Asp), CTAB, SDS, and polyvinyl alcohol (PVA) as a binary system of surfactants, was usually only a stencil effect [32,33,34,35], which played a certain role in regulating the morphology and size of n-HA crystal growth. The effect on improving dispersion was poor, and the residues in the product were difficult to remove. Langroudi MM et al. [36] used PVP as a template and SDS as a surfactant to synthesize bone-like n-HA via the bionic method. The results demonstrated that polymers and surfactants as polymer capsules could appropriately control the size, shape, morphology, and dispersion of HA crystals. All samples displayed biological activity because they could form carbonate apatite and grow HA on its surface, and the 3-(4,5)-dimethylthiahiazo (-z-y1)-3,5-di-phenytetrazoliumromide (MTT) test showed that the samples had good biocompatibility. Shanthi PMSL et al. [37] utilized the electrostatic interaction between surfactants to categorize them into two types: double anions (cetrimide and SDS) and double cations (cetrimide and CTAB), with a weight ratio of 1:1 and a total concentration of 0.28 g/100 mL. An effective morphological adjustment was performed on the samples. FTIR, XRD, FESEM, HRTEM, TGA/DTA, and BET analyses showed that the samples exhibited HAp phase with nano-scale and mesoporous properties. Anionic surfactants promoted the growth of the particles from spherical to hexagonal rods, while a mixture of double cations inhibited growth and led to disc-shaped HAp. The Ca^2+^ ion release assay of the sample showed that the biological activity of disc-shaped HAp was better than that of commercial HAp. Tari NE et al. [38] used the mixture of CaCl_2_ and H_3_PO_4_ (aqueous phase), the cationic surfactant CTAB, and the anionic sodium dodecyl to prepare n-HA particles with various shapes via the precipitation method. These surfactants formed various aggregates as templates in a mixture of rich cation and anionic regions. The results indicated that the morphology of HAP nano-particles could be controlled by changing the ratio of cationic and anionic surfactants in the mixture to synthesize HA nano-particles with high crystallinity and minimal agglomeration. Shanthi PMSL et al. [39] reported a successful preparation of shell-shaped nano-HAp spheres with a well-defined morphology, a uniform size of approximately 200 nm, and a stoichiometric ratio of 1.7 using the surfactant tetradecyltrimethylammonium bromide (cetrometin). Ma XY et al. [40] accomplished the synthesis of spherical n-HA with outstanding uniformity and regularity via the water-in-oil-microemulsion method at room temperature in a short duration, and span-80, cyclohexane, and Ca(NO_3_)_2_·4H_2_O and (NH_4_)_2_HPO_4_ solution were used as surfactants, oil phase, and water phase, respectively. The effects of the water–oil ratio and water–surfactant ratio on the stability of the micro-lotion system were studied, and a stable reaction system was established with proposed growth mechanisms. Yang L et al. [41] proposed a simple and mass synthesis route of HA nano-crystals with no agglomeration, excellent crystallization, and low aspect ratio. An improved co-precipitation process was utilized, and non-toxic gelatinized starch was used as the matrix without any other surfactant. This synthetic pathway had the potential to expand production scale, and the product had the same biocompatibility and biological activity as conventional n-HA. It also had the capability to produce other precipitated ceramic nano-particles with significantly reduced agglomeration and aspect ratio. Suslu A et al. [42] investigated the effect of surfactant types on the biocompatibility of electrospun HAp/poly(3-hydroxybutyrate-co-3-hydroxyvalerate) (PHBV) composite nano-fibers, and non-ionic Tween 20 and 12-hydroxysteric acid (HSA), cationic dodecyl trimethyl ammonium bromide (DTAB), and anionic sodium deoxycholate and SDS surfactants were used for comparison. The results indicated that the incorporation of HAp and any of the surfactant types strongly activated the precipitation rate of the apatite-like particles and decreased the percentage crystallinity of the HAp/PHBV mats.

According to the above-mentioned literature, it is evident that the introduction of surfactant into the preparation process of n-HA would improve the dispersion of n-HA.

### 3.4. Surface Modification by Grafting Polymer

**Figure 4:**
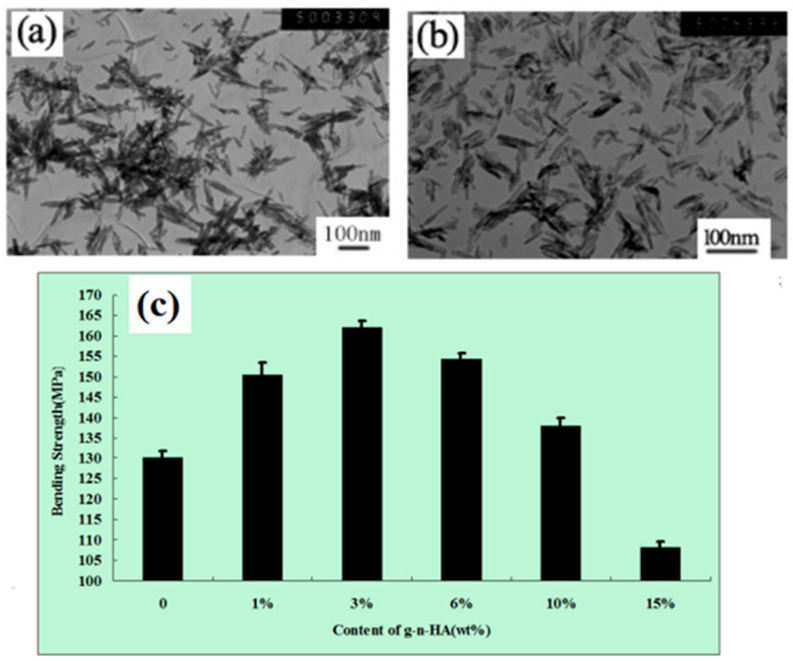
TEM photographs of (**a**) n-HA, (**b**) g-n-HA, and (**c**) bending strength of g-n-HA/PLGA composites with different contents of g-n-HA [53].

In order to improve the interfacial adhesion between n-HA and polymers, researchers have used various organic small molecules to coat them to reduce the surface energy of n-HA, such as silane coupling agents, isocyanates, fatty acids, tartaric acid, etidronic acid, polyhedral oligomeric silsesquioxanes, etc. [43,44,45,46,47,48,49,50]. Even various means of direct and indirect surface grafting or atom transfer radical polymerization grafting of polylactic acid were used to improve their interfacial adhesion [51], but the coating rate or grafting rate was relatively low, the modification effect was minimal when high-content n-HA was added, and the grafting process was cumbersome, toxic, and costly. In our previous studies [52,53,54,55], our research group also investigated various new methods for the combined grafting of lactide with some small organic molecules, such as stearic acid, citric acid, lysine, 3-amino propyltriethoxy silane (KH550), etc. The aforesaid modification methods had certain effects, which could improve the dispersion according to the TEM photographs of the before and after modification of n-HA, but the bending strength of the composite was significantly reduced compared to pure Poly(lactic-co-glycolic acid) (PLGA) when modified n-HA was added at the amount of over 15 wt% (as shown in Figure 4) because of the low grafting amount. Therefore, other modification methods need to be explored to solve the problem of n-HA/hydrophobic polymer composites.

Tang M et al. [56] used citric-acid-grafted surface-modified hydroxyapatite (SHA) to prepare SHA/GO composite materials via solution mixing and hydrothermal methods assisted by ultrasound. Before and after immersion in simulated body fluid (SBF) solution, the composite could effectively promote the mineralization of bone-like apatite. In vitro drug release tests showed that the change of GO content had a great influence on the adsorption performance of aspirin. It was expected that these SHA/GO composites could be used for biopharmaceutical loading. Polymer additives have recently been successfully applied to the surface modification and effective regulation of the morphology of various inorganic minerals. By encapsulating organic molecules to improve interfacial compatibility with polymers, one representative polymer type was an amphiphilic block copolymer, which was composed of two different hydrophilic segments. One segment exhibited strong interaction with inorganic ions or solids, whereas the other segments only played a dispersing or solubilizing role. The co-polymers could form a firm anchor on the surface of the inorganic compound, and the extended chain segments were stretched into the solvent. Liao L et al. [57] used surface grafting polymerization (γ-Benzyl-L-glutamic acid) on n-HA (PBLG-g-HA), and a novel PBLG-g-HA/Poly-L-lactic acid (PLLA) nano-composite was obtained. By chemically modifying the surface of HA, the uniform dispersion of n-HA in chloroform solution was effectively improved, achieving a transition from hydrophilicity to hydrophobicity on the surface of HA, thereby enhancing the interaction between HA and the PLLA matrix. Pei F et al. [58] prepared modified n-HA by grafting polydopamine (PDA), which was added to the polycaprolactone (PCL) matrix to enhance their interface bonding in the bone scaffold via selective laser sintering (SLS). The tensile strength and compressive strength of the scaffold were increased by 10% and 16%, respectively. Additionally, the scaffold exhibited favorable biological activity and cell compatibility, which could accelerate the formation of the apatite layer and promote cell adhesion, proliferation, and differentiation.

Park SJ et al. [59] synthesized n-HA grafted with L-glutamic acid and fixed it on Ti disc implants modified by albumin. Compared with the original titanium implant, the modified titanium implant enhanced the adhesion, proliferation, and cell viability of MC3T3-E1 cells, and the enhancement would facilitate the bone integration between the Ti implant and the dental bone. Mehri A et al. [60] prepared tyramine-grafted hydroxyapatite through the hydrothermal reaction. Tyramine was in situ grafted onto the surface of HAp to inhibit crystal growth by forming organic–inorganic hybrid nano-particles, thereby developing a multi-functional surface to ensure good compatibility with the surface of cell-modified HAp. Timpu D et al. [61] used arginine (Arg) or polyethyleneimine (branched BPEI, or linear LPEI) as a cationic modifier and dispersant to obtain functionalized nHAp through wet chemical technology. The results demonstrated that the prepared nano-particles had needle or plate shapes. Both Arg and PEI were successfully grafted onto nHAp, and LPEI-functionalized nHAp displayed good similarity with biological apatite and the best DNA binding capacity. When nHAp/LPEI nano-particles were incorporated into the porous matrix based on collagen/dimethylsilanediol hyaluronate, the compression modulus of the biological composite material was six times higher than that of the pure polymer matrix, and the composite sponge possessed high toughness in five consecutive compression tests without any permanent deformation and cracks. The aforementioned findings indicate that nHAp/LPEI nano-particles can be considered as promising materials for biomedical applications, functioning as gene carriers or reinforcing fillers with strong interfacial adhesion in bone engineering biological composites.

Mirhosseini MM et al. [62] synthesized functional HA nano-particles (HA-F127) by fixing Planck F127 on HA nano-particles. The F127 graft chain on the surface of HA formed a core–shell structure, which reduced the agglomeration of the modified nano-particles and improved the dispersity. Due to the excellent chain entanglement and interfacial crystallization of the modified HA in the polymer substrate, HA-F127 and unmodified HA were introduced into the PCL/P123 electrospun substrate, resulting in a nano-composite containing 4 wt% nano-fillers. Based on the strong interfacial adhesion between the fillers and the matrix, the molecular dynamics simulations confirmed that the strong interfacial interactions between HA-F127 and PCL/P123, HA-F127/PCL/P123 secured excellent mechanical properties, crystallinity percentage, and thermal stability. Therefore, the HA-F127/PCL/P123 nano-fiber scaffold was considered as a promising candidate for tissue engineering applications. Ma R et al. [63] used the silane-coupling agent KH560 for the grafting modification of bioactive HA particles and prepared an HA/polyether ether ketone (PEEK) composite through hot pressing. The results indicated that KH-560 was successfully modified HA(m-HA), and the tensile strength of the m-HA/PEEK composite reached its maximum value when the HA content was 5 wt%, which was 23% higher than that of the pure PEEK sample. In vivo biomechanical testing of m-HA/PEEK revealed that the growth of bone tissue around the m-HA/PEEK composite with 5 wt% HA content was better than that of specimens with different HA contents. The above results indicated that the bioactive filler HA had a nano-scale effect in the PEEK matrix, which is clearly corroborated by the growth of surrounding bone tissue in the body.

**Figure 5:**
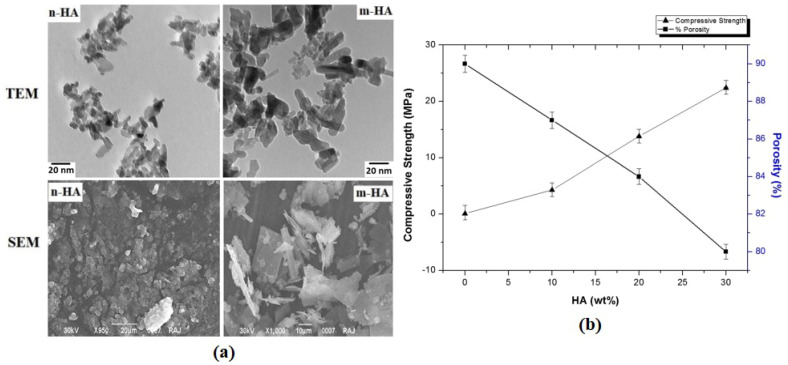
(**a**) TEM and SEM images of n-HA and modified n-HA; (**b**) comparison between porosity and compressive strength of m-nHA/PU nano-composite with varying concentrations of m-nHA [65].

Kairalla EC et al. [64] modified the surface of hydroxyapatite nano-crystals (HAPN) by grafting a three-arm star poly(ε-caprolactone) (SPCL). The results of albumin (HSA) and fibrinogen (HFb) adsorption indicated that SPCL-g-HAPN exhibited resistance to HFb adsorption compared with unmodified HAPN. ZP and CA measurements indicated that the heterogeneous topological structure of SPCL-g-HAPN was caused by the presence of hydrophobic and hydrophilic regions on the surface of the nano-composite. The enzymatic degradation of cholesterol esterase and lipase demonstrated that the hydrolysis rate of SPCL-g-HAPN was very slow via comparison with the SPCL/HAPN mixture. In vitro biological study indicated that human osteoblast-like cells (MG-63) possessed normal cell morphology and could adhere and spread across the surface of SPCL-g-HAPN. Compared with pure HAPN or SPCL materials, higher overall cell proliferation was observed on the SPCL-g-HAPN scaffold. Kumar L et al. [65] developed a porous modified n-HA/polyurethane (m-HA/PU) nano-composite scaffold for bone tissue engineering by grafting etidronic acid (ETD, 0.1 M) onto the surface of n-HA particles and strengthening it into polyurethane scaffolds prepared via the foaming method. As seen in Figure 5, it can be observed that the surface of m-HA particles was completely transformed from a granular structure to a sheet structure with a size of 40 nm. Furthermore, the compressive strength of the obtained PU/m-HA nano-composites with 30% filler concentration was 22.4 MPa, with a required porosity of 80%, showing that the PU nano-composite scaffolds were well suited for its bone healing application. In addition, the results of in vitro soaking in SBF for 4 weeks showed partial surface hydrolysis, and the cell culture results show that m-HA/PU nano-composite scaffolds were very suitable for bone tissue engineering.

Yang WF et al. [66] modified HA nano-particles with dopamine and hexamethylene diamine, and PLLA was connected to HA nano-particles through the ammonolysis reaction. The PLLA-modified HA nano-particles were mixed with PLLA to form thermoplastic composites for 3D printing. Due to the high compatibility between the PLLA matrix and PLLA-modified HA nano-particles, 3D-printed PLLA/HA scaffolds displayed strong mechanical properties and good biocompatibility, enabling flexible strategies for manufacturing scaffolds for customized treatment of bone defects. Wang Y et al. [67] successfully used the surface-initiated reverse atom transfer radical polymerization (reverse ATRP) technology to modify HAP nano-particles with polymethyl methacrylate (PMMA). The peroxide initiator component was covalently linked to the surface of HAP through the surface hydroxyl group. The reverse ATRP of methyl methacrylate (MMA) was carried out from the initiator functionalized HAP. Subsequently, the end bromine group of the grafted PMMA initiated the ATRP of MMA. The HAP nano-particles grafted with PMMA exhibited excellent dispersion in MMA monomer, and the dispersibility of surface-grafted HAP and the compressive strength of HAP/PMMA composites were improved with the increase in the amount of grafted PMMA. Dai YF et al. [68] grafted poly (L-phenylalanine) onto the surface of n-HA through the ring opening polymerization (ROP) of L-phenylalanine N-carboxylic anhydride. By optimizing the reaction conditions, the grafting amount of poly (L-phenylalanine) on the surface of HA could be increased to a range of 20.26% to 38.92%, and the crystal structure of modified HA was almost the same as that of HA. MTT results demonstrated that modified HA had good biocompatibility, indicating that the modified HA could have a potential application in bone tissue engineering, and that the ROP is an effective surface modification method.

Ku KL et al. [69] studied the surface modification of n-HAP with ethylene glycol and PCL sequentially via a two-step ring opening reaction, and the affinity was improved between the polymer and ceramic interphases of PCL-grafted ethylene glycol-HAP (PCL-g-HAP) in PMMA; that is, PCL-g-HAP/PMMA not only increased the interfacial adhesion between the nano-particles and cement but also better promoted biological activity and affinity between the osteoblast cells and PMMA composite cement. These results meant that g-HAP and its use in a polymer/bioceramic composite had great potential to improve the functionality of PMMA cement. Furthermore, the composite of PCL-g-HAP with poly (1,6-bis-(p-carboxylphenoxyhexane) co-(sebacic anhydride)) (PANH) was studied. The PCL-g-HAP/PANH composite exhibited excellent mechanical properties and a rapid degradation rate. Preliminary in vivo studies for rat skull repair had affirmed the superior performance of the PCL-g-HAP/PANH composite, which had great potential to be a novel matrix for bone tissue engineering [70]. Zarif F et al. [71] synthesized citric acid and aspartic acid grafted HA (g-HA) via the in situ co-precipitation method and explored its controlled delivery of moxifloxacin. The results revealed that g-HA, characterized by high surface area and surface charge and low crystallinity, strengthened its electrostatic interaction with the antibiotic moxifloxacin and decreased the drug release in vitro compared with pure HA. The in vitro antibacterial activity manifested that the drug release of HA and g-HA was against *Staphylococcus aureus* and *Enterobacteriaceae*, and the MTT assay confirmed the biocompatibility of HA and g-HA. Li HB et al. [72] utilized HA nano-particles to graft onto the surface of polyethylenemethacrylate (PEGMA) and cross-link with polyethylene methacrylate (PEGDMA) under ultraviolet light to form a composite. The dispersion of HA g-PEGMA nano-particles in the poly (PEGDMA) matrix was better than that of n-HA. With a load of 1 wt%, the strength and modulus of the composite were increased by 14% and 9%, respectively.

Kumar L et al. [73] modified n-HA with triethanolamine (TEA-nHA), and the morphology of TEA-nHA was successfully changed from particles to irregular sheets/plates. Compared with pure (PU) composites, the PU/TEA-nHA nano-composite formed with castor oil-based PU with a content of 40 wt% possessed open and interconnected pores with a size range of 150–700 µm, and the compressive strength and porosity of the composites were 20.7 MPa and the porosity was ≤82%, respectively. The cellular compatibility of these new engineering surfaces could maintain exponential growth for up to 8 days and enhance cell viability. Overall, the developed surfaces had improved cell growth, suggesting that the PU/TEA-nHA nano-composite was capable of promoting bone tissue regeneration. Mehmanchi M et al. [74] successfully grafted the arm with the functional group of uracil pyrimidinone with a self-association ability through the tetrahydrogen bond onto the n-HA. Compared with the original n-HA, the supermolecular modified nano-particles (n-HAP-UPy) enhanced the colloidal stability, and they were uniformly dispersed in PCL with different filler loads. Preliminary cell results clearly confirmed that the supramolecular nano-composites were non-toxic and biocompatible. Pielichowska K et al. [75] studied a functionalized n-HAP with PCL, using 1,6-hexamethylene diisocyanate (HDI) as a coupling agent, and then incorporated it into the polyoxymethylene copolymer (POM) matrix using the extrusion technique to obtain POM/HAP-g-PCL composites. It was found that the introduction of HAP-g-PCL to the POM matrix had a limited effect on the phase transitions of POM and its degree of crystallinity, and it caused a significant increase in the thermal stability of the POM. In particular, the crucial parameter in biomedical applications, namely, the in vitro bioactivity, was improved, albeit slightly decreasing the mechanical properties of POM composites.

Zhang M et al. [76] used stearic acid to modify HAP in different solvents (water, ethanol, or dichloromethane (CH_2_Cl_2_)) and studied the effects of different solvents on the properties of HAP particles (activation rate, grafting rate, chemical properties), lotion properties (lotion stability, lotion type, droplet morphology), and cured materials (morphology, average pore size). The results confirmed that there was interaction between stearic acid and HAP particles, and the hydrophobicity of HAP particles was enhanced after surface modification. It was best to use ethanol as the solvent for stearic acid to modify HAP particles so that the stability of Pickering lotion could be improved and the cured samples with uniform pore size could be obtained. Song XF et al. [77] prepared PLLA-g-HA by adding ethylene-glycol-tethered hexamethylene diisocyanate. The results showed that the grafting ratio of HA was 25% higher than that obtained by unmodified HA or HA modified with L-lactic acid, and it could be stably dispersed in chloroform for more than 2 days. The tensile test after co-electrospinning fibers showed that the mechanical properties of the PLLA-g-HA/PLGA composite fiber membrane were higher than those of the HA/PLGA membrane. Jiang YR et al. [78] used a series of aminoalkyl phosphates (AAP-n, carbon atom number n between 2 and 6) as surface modifiers to prepare HA hydrolytic colloid. The obtained nano-particles (Cn-HA) had a core–shell structure, in which the ionized layer of calcium (AAP-n) complex [+H_3_N-(CH_2_)n-OPO_3_ Ca] encapsulated each HA core. Due to the electrostatic repulsion between suspended particles, long-term colloidal stability could be achieved. The introduction of AAP-n led to a particle aspect ratio increase from C2-HA to C6-HA along the c-axis of the crystal. Preliminary cell culture using osteoblast-like MG-63 cells showed no cytotoxicity associated with the prepared Cn-HA particles. The above results indicated that the functional amino groups around the nano-particles could be used to graft various organic chains to prepare homogeneous HA/polymer composites as bone-bonding materials. Wei JC et al. [79] proposed the surface modification of n-HA via the ring opening polymerization (ROP) of γ-benzyl-L-glutamate N-carboxyanhydride (BLG-NCA) to prepare PBLG-g-HA. The results showed that the PBLG-g-HA hybrid could form an interpenetrating net structure during the self-assembly process. The PBLG-g-HA hybrid could maintain higher colloid stability than the pure HA nano-particles, and the in vitro cell cultures suggested that the cell adhesion ability of PBLG-g-HA was much better than that of pure HA.

Makvandi P et al. [80] modified commercial HAP at the micron level with methacrylate and quaternary ammonium salts, and different amounts (i.e., 2.5, 5, and 10 wt%) were used as fillers for UV-cured custom resins in stereolithography (SLA). Compared with pure resins, all modified HA particles (m-HAP)-filled composite had higher strength, and the antibacterial activity of the composite increased with the increase in m-HAP content. Compared to pure HAP, the complex of m-HAP (i.e., 2.5%) exhibited sufficient antibacterial activity and reduced the growth of bacteria and fungi, even with low concentrations. All results summarized that samples containing 5% m-HAP could be considered as the best comprehensive solution for thermal, chemical physical, mechanical, and biological properties, and the composite was selected by SLA to construct an open bite prototype. Li K et al. [81] prepared HA nano-rods doped with Fe and Si on Ti, and the antibacterial peptide HHC-36 was chemically bonded to the nano-rods with and without a polymer brush as a gasket. The results showed that the grafting of polymer brushes onto HHC-36 did not substantially alter the microstructure of the nano-rods, but the brushes effectively increased the loading and stability of HHC-36. Moreover, with the assistance of HHC-36, the synergistic effect of adenosine monophosphate (AMP)-derived antimicrobial peptides and the physical puncture of HA nano-rods could effectively kill *Staphylococcus aureus*. Compared with Ti, the formation of biofilm was inhibited in phosphate buffer solution and nutrient-rich medium. HA nano-rods with polymer-brushed HHC-36 killed 99.5% of *Staphylococcus aureus* and 99.9% of *Escherichia coli*, and they exhibited cellular compatibility in vitro, inhibiting bacterial infections and reducing inflammatory reactions in vivo, which indicates that the polymer-brushed HHC-36 on HA nano-rods had enormous potential application on the Ti surface.

Tham DQ et al. [82] successfully prepared vinyl trimethoxysilane-treated HA (vHAP) and PMMA-grafted HAP (gHAP) using original HAP (oHAP) as the raw material. Three groups of HAP-modified PMMA bone cement (oHAP-BC, vHAP-BC, and gHAP-BC) were prepared using three HAPs (oHAP, vHAP, and gHAP) as additives. The results showed that the setting time of HAP-modified bone cement was longer, the maximum exothermic temperature was lower, and the vHAP and gHAP nano-particles were better dispersed in the polymerized PMMA matrix than oHAP nano-particles, thus meeting the requirement of the mechanical properties, which proved the effectiveness of organic functionally grafted HA in acrylic bone cement. Dorm BC et al. [83] studied two sources of L-alanine and three grafting methods for the surface functionalization of HA. The results showed that 8–25 wt% of organic matter was formed in HA. The viability of MG-63 human osteoblasts incubated with alanine grafted HA samples for 24 h was well preserved, which was higher than that of cells incubated with HA in all cases. Alanine-grafted HA prepared in situ and by simple mixing showed higher protein adsorption and cell adhesion, respectively, indicating that it was promising in regenerative medicine.

Elbasuney et al. [84] used poly (ethylene copolymerization AA) polymer surfactant to modify the surfaces of HA nano-plates. The surface properties of organic modified HA nano-plates changed from hydrophilicity to hydrophobicity. It demonstrated effective phase transfer from the aqueous phase to the organic phase, reducing the size of the nanoplate to 100 nm L and 50 nm W. By way of surface modification with dodecanedioic acid, layered HA plates were further developed. This method could provide laminated or peeled plates for effective integration into biocompatible polymers, giving hope for the green synthesis of hyaluronic acid nano-particles with controllable morphology and surface properties. Xu M et al. [85] obtained modified HA (HA-APS) with active amino groups on the surface via reaction with silane coupling agent KH-550, and then initiated L-aspartic acid-β-HA grafted with poly (benzyl aspartate) (PBLA) prepared via the ring opening polymerization of benzyl ester N-carboxylic anhydride (BLA-NCA), which realized the transition of the surface of HA from hydrophilicity to hydrophobicity. The dispersion experiment confirmed that the surface-modified HA by PBLA could significantly increase the hydrophobicity of the HA surface and prevent the aggregation of nano-HA particles. Heng CN et al. [86] developed a simple surface-initiated polymerization strategy for n-HA via combination of the surface ligand exchange and reversible addition fragmentation chain transfer (RAFT) polymerization to improve the dispersibility in aqueous solution, where HA nano-rods were first modified with riboflavin-5-phosphate sodium (RPSSD) via ligand exchange reaction between the phosphate group of RPSSD and oleic acid. Then, the hydroxyl group of nHAP-RPSSD was used to immobilize the chain transfer agent, which was used as the initiator for surface-initiated RAFT polymerization. Results showed that nHAP-RPSSD-poly(IA-PEGMA) exhibited excellent water dispersibility, desirable optical properties, good biocompatibility, and high drug loading capability, making it a promising candidate for biological imaging and controlled drug delivery applications in bone repair fields.

According to the results of the listed literature, we think that surface grafting polymers onto n-HA is an effective method, which could improve not only the dispersion of n-HA but also the interfacial adhesion between n-HA and polymers. Moreover, the higher grafted amount would be more conducive to enhancing the mechanical properties of nano-composite.

### 3.5. Preparation of Hybrid Nano-Apatite by Introducing Macromolecule

**Figure 6:**
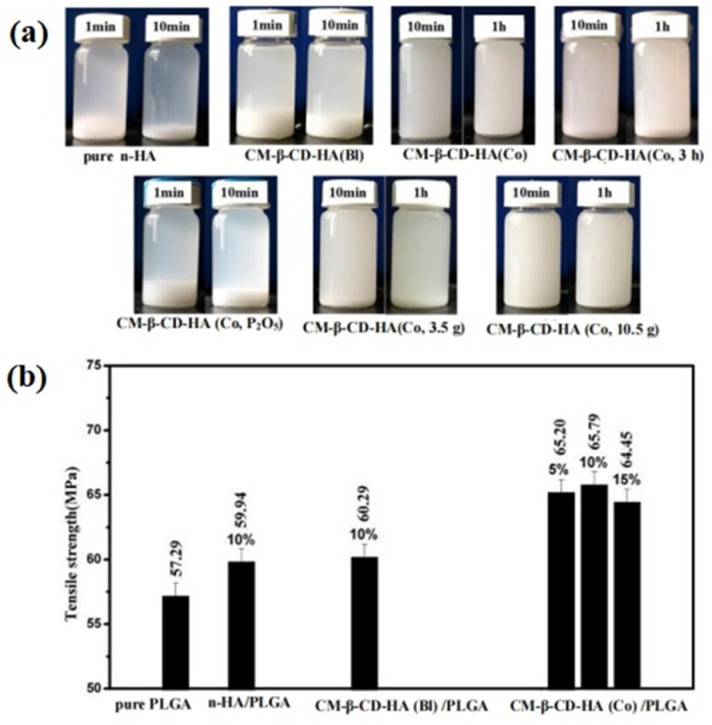
(**a**) Dispersion pictures of nano-particles in dichloromethane for different time points; (**b**) tensile strength of samples [90].

To obtain n-HA with excellent dispersion, Zhang P et al. [80] paid attention to the raw materials of preparation n-HA, where polyethylene glycol monomethyl ether phosphate (P-MPEG) was used as the auxiliary phosphorus source and steric hindrance, and the hybrid nano-apatite that extended the MPEG chain beyond the n-HA crystal structure was prepared via the co-precipitation method, which could not only be dispersed in water but also in organic solvents such as methanol and dimethylformamide (DMF). Obviously, the hybrid nano-apatite prepared from the raw materials could effectively raise its dispersion and compound with water-insoluble polymers owing to the change in surface properties via the introduction of the MPEG hydrophobic structure chain. However, the molecular weight of P-MPEG, selected in this paper, was very small, and its steric hindrance and hydrophobicity improvement were very limited. It was still necessary to explore the preparation of hybrid nano-apatite by introducing other amphiphilic macromolecules. Cyclodextrin has a unique amphiphilic structure, which is hydrophobic in the cavity and hydrophilic outside the cavity. Cyclodextrin macromolecules are functional materials in bone materials. In the preparation of HA, it was reported that there was a certain chemical bonding interaction between HA and cyclodextrin macromolecules [88,89]. Therefore, in our research group [90,91], we explored the influence of cyclodextrin macromolecules of different sorts, addition orders, reaction times, addition amounts, and other factors on the preparation, structure, and dispersion of hybrid nano-apatite (shown in Figure 6). According to Figure 6, it can be confirmed that the dispersion of carboxylated cyclodextrin hybrid nano-apatite (CM-β-CD-HA(Co)) was significantly augmented, and the tensile strength of the composite with the additional amount of 10 wt% CM-β-CD-HA(Co) was the best, which was 14.84% higher than that of pure PLGA. The results confirmed that the hybrid nano-apatite obtained through a new surface modification strategy has significant potential as a reinforcement filler for PLGA used as bone materials in the future.

In addition, as we know, with the increasing consumption of non-renewable resources such as oil and coal and the rapid rise in raw material prices, the hybrid nano-apatite has attracted widespread attention for the development and utilization of green and environmentally friendly natural resources. Lignin is the only plant resource containing a benzene ring structure in nature, and it is non-toxic, biodegradable, biocompatible, and possesses some special properties, such as antibacterial, antioxidant, and UV absorption functions, so it is an ideal raw material for preparing functional materials. Ho YK et al. [92] reported that lignin could act as a gene carrier by forming complexes with DNA after co-polymerization with other polymers, and it displayed a high infection rate and low cytotoxicity. Although the above literature indicated that lignin was an excellent green and environmentally friendly chemical raw material, and it was non-toxic to organisms, there have been no reports on lignin being used for the modification of n-HA. Therefore, in our recent research [93], we explored the preparation of hybrid nano-apatite by introducing lignin, adopting the co-precipitation method, and the obtained hybrid nano-apatite displayed excellent dispersion and promoted crystallization effects, which could greatly improve the mechanical strength of PLGA. In addition, in vitro cell culture experiment results indicated that lignin surface hybridization of n-HA was beneficial for improving the cell biocompatibility of PLGA, suggesting that the introduction of lignin was a novel method for obtaining highly dispersed n-HA, and it would provide a new idea for the future implementation of n-HA/PLGA nano-composites as bone materials and offer a new means for application of lignin in the biomedical field. Subsequently, our research team [94] also explored the preparation of a new hybrid nano-apatite via the co-hybrid of lignin and cyclodextrin (g1-HA). The results showed that the hybrid of lignin and cyclodextrin for n-HA had an excellent synergistic effect, which could improve the dispersion, and produced good interface bonding between hybrid nano-apatite and PLGA matrix. When the amount of hybrid nano-apatite was 15 wt%, the tensile strength of the composite was still 14.53% higher than that of PLGA, which was significantly better than the hybrid nano-apatite with lignin or cyclodextrin alone. In addition, the results of immersion in SBF and in vitro cell experiments showed that the co-hybrid nano-apatite had good degradation performance, apatite deposition, and excellent cell biocompatibility. This study could provide important guidance for obtaining a highly dispersed n-HA as a PLGA-based reinforcing filler for bone materials.

## 4. Conclusions

In summary, n-HA particles have great application in the bone materials field. The research on the synthesis method and surface modification of n-HA has made some progress, and its modification effect has its own emphasis; for example, ion doping usually improves the biological activity significantly, while the template method can regulate its morphology and adsorption performance. Grafting small molecules or polymers can optimize surface characteristics and interface bonding with polymers, while hybrid nano-apatite, by introducing amphiphilic macromolecules, can significantly improve dispersion, and it has a great promoting effect for the application of n-HA. In future research, we think the following aspects should be considered: (1) more transition metals and some rare earth ions should be designed to substitute Ca^2+^ so as to endow n-HA with some new properties, such as luminescence, magnetism, conductivity, etc., which would broaden its application in biomedical fields beyond bone materials, including the diagnosis and treatment of diseases, especially cancer; (2) it is necessary to combine the introduction of ion doping with functional small molecules or polymers during the preparation of n-HA so as to obtain multi-functional hybrid nano-apatite with high dispersion and good biological activity; (3) some key technologies for controlling the size and morphology of n-HA particles should be deeply studied so as to extend their applications in various fields; (4) theoretical calculation by means of quantum chemistry for the structure of modified n-HA should be emphasized so that some properties of the modified n-HA—for example, the changes in surface properties, the dispersion improvement, and so on—can be further explained. This could also help predict the effective modification methods so as to obtain a more ideal surface state. To summarize, we believe that meaningful surface modifications of n-HA will be developed in the future, which would expand the application of n-HA particles in the biomedical field.

## Figures and Tables

**Table 1:** Modification methods of n-HA.

Modification of n-HA	
Physical Modification	Chemical Modification
Physical adsorption	Template method
Electron induction	Ion doped (Single ion doped, Multiple ions co-doped)
Laser irradiation	Adding surfactants for modification
	Surface modification by grafting polymer
	Preparation of hybrid nano-apatite by introducing macromolecule

**Table 2:** Comparison of the different chemical modification methods.

	Methods	Modification Effect	References
Physical modification	Physical adsorption, electron induction, and laser irradiation	Improve the stability and dispersion of nano-particles	[9,10]
Chemical modification	Template method	The modified n-HA with different morphologies or structures is obtained by removing the template	[11,12,13,14]
	Ion doped	Adding corresponding ions to the reactants to alter the surface characteristics of n-HA	[15,16,17,18,19,20,21]
	Adding surfactants for modification	Adsorption of their groups of surfactants on the n-HA particle surface	[22,23,24,25,26,27,28,29,30,31,32,33,34,35,36,37,38,39,40,41,42]
	Surface modification by grafting polymer	Some polymers were grafted onto n-HA to improve the interface adhesion between n-HA and polymers	[43,44,45,46,47,48,49,50,51,52,53,54,55,56,57,58,59,60,61,62,63,64,65,66,67,68,69,70,71,72,73,74,75,76,77,78,79]
	Preparation of hybrid nano-apatite by introducing macromolecule	Some amphiphilic macromolecules were introduced to obtain hybrid nano-apatite, which displayed better reinforce effect for polymers	[80,81,82,83,84,85,86,87,88,89,90,91,92,93,94]

## Data Availability

The data that support the findings of this study are rearranged from the reported references and available within the article.

## Abbreviations

BLA-NCA	Benzyl ester n-carboxylic anhydride
BLG-NCA	γ-benzyl-l-glutamate n-carboxyanhydride
ChS	Chondroitin sulfate
DMF	Dimethylformamide
ETD	Etidronic acid
PCL-g-HAP	PCL-grafted ethylene glycol-HAP
HAPN	Hydroxyapatite nano-crystals
HDI	1,6-hexamethylene diisocyanate
HFb	Fibrinogen
LE	Licorice root extract
m-HA	Modified HA particle
oHAP-BC	Original HAP bone cement
vHAP-BC	Vinyl trimethoxysilane treated HA bone cement
gHAP-BC	HAP-modified PMMA bone cement
PANH	Poly (1,6-bis-(p-carboxylphenoxyhexane) co-(sebacic anhydride))
PBLA	Poly (benzyl aspartate)
PHBV	poly(3-hydroxybutyrate-co-3-hydroxyvalerate)
PBLG-g-HA	Surface grafting polymerization (γ-benzyl-l-glutamic acid) on n-HA
PEEK	Polyether ether ketone
PEGMA	Poly ethylene methacrylate
PMMA	Polymethyl methacrylate
P-MPEG	Polyethylene glycol monomethyl ether phosphate
POM	Polyoxymethylene copolymer
PVP	Polyvinylpyrrolidone
RAFT	Eversible addition fragmentation chain transfer
ROP	Ring opening polymerization
RPSSD	Riboflavin-5-phosphate sodium
DTAB	Dodecyl trimethyl ammonium bromide
SDBS	Sodium dodecyl benzene sulfonate
SDS	Sodium dodecyl sulfate
SHA	Surface-modified hydroxyapatite
SLS	Selective laser sintering
SPCL	Poly(ε-caprolactone)
AMP	Adenosine monophosphate
Asp	Aspartic acid
ATRP	Atom transfer radical polymerization
CTAB	Cetyltriethylammonium bromide
CTS	Chitosan
Gly	Glycine
GO	Graphene oxide
HA, HAP	Hydroxyapatite
HSA	Albumin
KH550	3-aminopropyltriethoxysilane
MTT	3-(4,5)-dimethylthiahiazo (-z-y1)-3,5-di- phenytetrazoliumromide
n-HA	Nano-hydroxyapatite
PARP	Poly (ADP-ribose) polymerase
PCL	Polycaprolactone
PDA	Polydopamine
PEI	Polyethyleneimine
PET	Polyethylene terephthalate
PLGA	Poly(lactic-co-glycolic acid)
PLLA	Poly-L-lactic acid
PU	Polyurethane
PVD	Polyvinylpyrrolidone
SBF	Simulated body fluid
Sr-HA	Strontium substituted hydroxyapatite
